# Allicin Attenuates Sepsis‐Induced Acute Kidney Injury by Inhibiting Pyroptosis Through Negative Regulation of the PI3K/AKT Pathway

**DOI:** 10.1155/mi/5454020

**Published:** 2026-04-09

**Authors:** Yanlei Zheng, Shi Li, Li Zhu, Li Zhang

**Affiliations:** ^1^ Department of Critical Care Medicine, Hubei Cancer Hospital, Tongji Medical College, Huazhong University of Science and Technology, Wuhan, 430079, Hubei Province, China, hust.edu.cn

**Keywords:** acute kidney injury, allicin, PI3K/AKT pathway, pyroptosis, sepsis

## Abstract

Pyroptosis is a critical mechanism leading to sepsis‐induced acute kidney injury (S‐AKI). Allicin, an organosulfur compound extracted from garlic bulbs, protects against S‐AKI through its anti‐pyroptotic role, but the underlying pathophysiological mechanisms remain largely unknown. In in vivo and in vitro experiments, rats and HK‐2 cells were induced by caecal ligation and puncture (CLP) and lipopolysaccharides (LPSs), respectively. They were then treated with allicin, insulin‐like growth factor‐1 (IGF‐1, a PI3K/AKT agonist), or both. In this study, we found that the levels of p‐PI3K, p‐AKT, NF‐κB, NLRP3, caspase‐1, gasdermin D (GSDMD)‐N, IL‐1β, IL‐18 and lactate dehydrogenase (LDH) were significantly increased, which was accompanied by kidney tissue and HK‐2 cell pyroptosis, eventually leading to elevated serum creatinine (Scr) and blood urea nitrogen (BUN) levels, increased 7‐day mortality and reduced HK‐2 cell activity in CLP‐induced rats and LPS‐induced HK‐2 cells, respectively. Moreover, we observed that allicin significantly reduced the levels of p‐PI3K, p‐AKT, NF‐κB, NLRP3, caspase‐1, GSDMD‐N, IL‐1β, IL‐18 and LDH and inhibited pyroptosis in renal tissues and HK‐2 cells, ultimately resulting in improved kidney function, 7‐day survival, and enhanced HK‐2 cell activity in CLP‐induced rats and LPS‐induced HK‐2 cells. Furthermore, our results demonstrate that agonism of the PI3K/AKT pathway—by using IGF‐1—could reverse the abovementioned protective role of allicin, accompanied by increased phosphorylation of PI3K and AKT in vivo and in vitro. Overall, the findings of this study demonstrate that the protective effect of allicin on S‐AKI is largely dependent on the inhibition of pyroptosis through the negative regulation of the PI3K/AKT pathway.

## 1. Introduction

According to the World Health Organization (WHO), sepsis is a global health priority [1]. Sepsis‐induced acute kidney injury (S‐AKI) is a common complication of sepsis and is most commonly seen in hospitalised elderly patients [2–4]. Studies have reported that ~40%–50% of sepsis patients in intensive care units develop AKI [5], with a mortality rate of ~40% [6]. Despite the continuous progress in clinical treatment methods, the mortality rate of S‐AKI patients remains high, which is attributed to its unclear pathogenesis. In the past, it was believed that the occurrence of S‐AKI might be related to renal hypoperfusion, excessive inflammation, apoptosis, oxidative stress, etc. [7–9]. Existing evidence also indicates that renal tissue pyroptosis during sepsis is a critical event in the development of S‐AKI [10, 11]. In recent years, with the advances in pyroptosis research, exploring its mechanisms has become valuable for understanding bacterial infection, sepsis, and other related processes [12].

Allicin is a type of volatile oil extracted from garlic. A chemical compound called diallyl thiosulfinate exhibits a variety of biological activities, such as the protection of heart and kidney function and antibacterial, anti‐inflammatory, and anti‐tumour activities [13]. Currently, allicin is widely studied in sepsis‐related injuries [14], but its effect on pyroptosis in sepsis has not been reported. Pyroptosis is a new type of programmed inflammatory cell death that differs from apoptosis and necrosis. Pyroptosis occurs more rapidly and is accompanied by the release of numerous pro‐inflammatory factors [15, 16]. Renal tubular epithelial cell injury, in which pyroptosis plays a role in AKI progression, is a characteristic pathological change [17].

Interestingly, during sepsis, pathogens invade and release pathogen‐associated molecular patterns (PAMPs), such as lipopolysaccharides (LPSs), which bind to body pattern recognition receptors (PRRs) to trigger the onset of the host inflammatory response [18, 19]. Following initiation of this process, NLRP3 (a critical member of the nod‐like receptor family)‐mediated activation of caspase‐1 leads to cleavage of gasdermin D (GSDMD) into an active N‐terminus and formation of cell membrane pores. This process promotes the production and release of pro‐inflammatory mediators, such as IL‐1β and IL‐18, ultimately forming the classical pathway of cellular pyroptosis [20, 21]. Phosphoinositide 3‐kinase/protein kinase B (p‐PI3K/AKT) promotes the translocation of active NF‐κB into the nucleus, which ultimately leads to pyroptosis and inflammatory responses in chondrocytes through the activation of NLRP3 [22]. In a mouse osteoarthritis model, allicin exerted an anti‐inflammatory effect and alleviated disease progression by inhibiting the PI3K/AKT/NF‐κB pathway [23]. However, whether allicin affects pyroptosis in S‐AKI by regulating the PI3K/AKT pathway remains largely unknown.

In this study, we hypothesised that allicin plays a protective role against S‐AKI. To test this hypothesis, we established rat and HK‐2 cell models of sepsis using caecal ligation and puncture (CLP) and LPS, respectively, and investigated the effect of allicin on S‐AKI through the inhibition of pyroptosis via regulation of the PI3K/AKT pathway.

## 2. Materials and Methods

### 2.1. Materials

Allicin (Number: A875877) was purchased from Shanghai Maclin Biochemical Technology Co., Ltd. (China). Recombinant human insulin‐like growth factor‐1 (IGF‐1) (AF‐100−11, a PI3K/AKT agonist) and recombinant rat IGF‐1 (P6371) were obtained from PeproTech (USA) and Beyotime (China), respectively. LPS was purchased from Sigma–Aldrich (Shanghai) Trading Co., Ltd. Male Sprague‒Dawley (SD) rats and HK‐2 cells were obtained from HY Cell Biotechnology (Wuhan, China). PI3K, p‐PI3K, AKT, p‐AKT, NF‐κB (p65), NLRP3, caspase‐1 and N‐GSDMD primary antibodies were purchased from Abcam (USA). A FAM‐FLICA caspase‐1 kit was obtained from Immunochemistry Technologies (Bloomington, MN, USA). IL‐1β and IL‐18 were purchased from Beyotime (China), and the lactate dehydrogenase (LDH) kit was obtained from Nanjing Jiancheng Biology (China).

### 2.2. Animals, Cells and Modelling

SD rats (*n* = 100, weighing 220–280 g) were obtained from the Center of Experimental Animals of Wuhan University in China. The rats were housed in a controlled environment (12 h light/dark cycle, temperature 25°C, humidity 50%) with free access to food and water for 7 days before the experiments. The rats were randomly divided into four groups (*n* = 25). All rats underwent complete survival observation. For biochemical analyses (serum creatinine [Scr], blood urea nitrogen [BUN], IL‐1β and IL‐18 levels) and western blotting, 15 rats per group were used for sample collection; the remaining 10 rats per group were used to validate the reproducibility of the results. This design ensured adequate statistical power (expected effect size *d* = 0.6, *α* = 0.05, power = 80%). which was calculated using 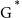
Power software (Version 3.1.9.7) for a two‐tailed test, confirming that a sample size of 15 per group for biochemical analyses was sufficient to detect the specified effect size. A rat sepsis model was constructed by CLP [24]. Rats were injected intraperitoneally with 1% sodium pentobarbital at a dose of 50 mg/kg (dissolved in saline) and then sacrificed by cervical dislocation before the relevant specimens were obtained. In addition, the control group underwent only exploratory laparotomy without ligation. Animal experiments were conducted in accordance with the regulations approved by the Ethics Committee of Hubei Cancer Hospital, affiliated with Tongji Medical College of Huazhong University of Science and Technology. Rats were treated with allicin (30 mg/kg, dissolved in saline, intraperitoneal [IP]) [25], IGF‐1 (5 μg/kg, dissolved in saline, IP), or both, to study the effect of allicin on CLP‐induced S‐AKI in vivo. HK‐2 cells (cell line: HK‐2 cells (human renal proximal tubular epithelial cells; species: *Homo sapiens*; tissue of origin: renal proximal tubule; official name: HK‐2; RRID: CVCL_0040) were purchased from the BeNa Culture Collection (China) in March 2023. HK‐2 cells were cultured in DMEM (Gibco, USA) supplemented with 10% foetal bovine serum (Gibco, USA). Experimental cell models were constructed by inducing HK‐2 cells with LPS (10 μg/mL, dissolved in DMEM). In the control group, cells were merely cultured under standard conditions and did not undergo any treatment. Cells were treated with allicin (50 mg/L, dissolved in DMEM) [26], IGF‐1 (100 ng/mL, dissolved in DMEM), or both, to investigate the effect of allicin on HK‐2 cell damage induced by LPS (serotype O111:B4) in vitro. Note: Cells were tested for mycoplasma contamination using the MycoAlert assay (Lonza) before each experiment, and all results were negative.

### 2.3. HK‐2 Cell Viability Assay

HK‐2 cells were treated with allicin and/or IGF‐1, followed by LPS for 24 h, and analysed using a cell counting kit‐8 (CCK‐8) according to the manufacturer’s instructions.

### 2.4. The 7‐Day Survival Rate

Sepsis was induced in the rat model using the CLP method, which marked the starting point of the experiment (Day 0). No interventions were applied during the initial 24‐h period post‐induction. At the 24‐h mark (Day 1), the CLP + A group received allicin (30 mg/kg, IP), the CLP + A + I group received allicin + IGF‐1 (30 mg/kg + 5 μg/kg, IP), and the control group received saline solution. Rats were then observed for 7 days following allicin administration, and survival was recorded at regular intervals.

### 2.5. LDH Assay

According to the instructions of the LDH kit, the supernatant of each group was collected, and the LDH release rate of the HK‐2 cells in each group was measured. The remaining adherent cells were subsequently lysed to determine the LDH levels, and the sum of the two values was considered the total LDH level.

LDH release rate%= LDH in the supernatant/LDH in the supernatant+LDH in the lysed cells×100%.

### 2.6. Detection of Scr, BUN, IL‐1β and IL‐18 Levels by ELISA

Serum samples from rats in each group were collected to determine the levels of Scr, BUN, IL‐1β and IL‐18 and the latter for IL‐1β and IL‐18 levels according to the manufacturer’s instructions.

### 2.7. Western Blot Analysis

The expression levels of p‐PI3K, PI3K, p‐AKT, AKT, NF‐κB (p65), NLRP3, caspase‐1 and GSDMD‐N in vivo and in vitro were measured by western blotting. Total protein from kidney or HK‐2 cells was extracted using RIPA lysis buffer, and protein concentration was measured by a bicinchoninic acid assay. Samples from each group were separated on an SDS‒polyacrylamide gel and transferred to PVDF membranes. After blocking with 5% PBS, membranes were incubated with primary antibodies (anti‐p‐PI3K, 1:1000 dilution; anti‐PI3K, 1:1000 dilution; anti‐p‐AKT, 1:1000 dilution; anti‐AKT, 1:1000 dilution; anti‐NF‐κB, 1:1000 dilution; anti‐caspase‐1, 1:800 dilution; anti‐GSDMD‐N, 1:800 dilution) overnight on a shaker at 4°C. Then, the PVDF membranes were washed with tris‐buffered saline and Tween (TBST) buffer solution and incubated with secondary antibody (LI‐COR, Lincoln, NE, USA; 1:5000 dilution) for 1.5 h at room temperature. Finally, the membranes were detected using a LI‐COR Odyssey Infrared Imaging System (LI‐COR Biosciences Inc., USA).

### 2.8. Haematoxylin–Eosin (HE) Staining of Kidney Tissue

Kidney tissues from each group were collected and fixed with 4% formaldehyde solution at room temperature for 5 min, embedded in paraffin, and cut into 3 μm‐thick sections. When sections completed HE staining, they were scored according to the degree of renal histological damage, including oedema, tubular dilation, haemorrhage and vacuolar degeneration of tubular epithelial cells (five‐score: 1, histopathological changes <10%; 2, = 10%–25%; 3, = 25%–50%; 4, = 50%–75%; and 5, >76%) [27]. Note: Pathological scoring of the HE‐stained sections was performed by two blinded pathologists (ICC = 0.89), with samples labelled randomly.

### 2.9. Pyroptosis in Kidney Tissues Was Detected by Terminal dUTP Nick Endlabelling (TUNEL) and Caspase‐1 Immunofluorescence Double Staining

Renal tissue of each group was fixed in 4% paraformaldehyde, frozen, added to the appropriate reagent 1 (TdT) and reagent 2 (dUTP) and streptavidin‐FITC‐labelled working solution, incubated with primary antibody (caspase‐1, 1:100) overnight at 4°C, incubated with II antibody (anti‐rabbit‐IgG labelled fluorescent antibody, 1:50), stained with 4^′^, 6‐diamidino‐2‐phenylindole (DAPI) fluorescent dye working solution at 37°C, and sealed with glycerol in a dark chamber. Next, TUNEL (green) and caspase‐1 (red) staining were observed under a fluorescence microscope; the co‐expressing cells (green and red overlap) exhibited pyroptosis.

### 2.10. Pyroptosis of HK‐2 Cells Was Detected by Propidium Iodide (PI) Staining

Cells from each group were collected and fixed with 4% paraformaldehyde for 30 min, washed three times with PBS, and incubated with PI staining solution for 30 min at 37°C in the dark. After the PI staining solution was removed, cells were stained with DAPI for 5 min and washed with PBS three times, after which the staining was observed under a fluorescence microscope.

### 2.11. HK‐2 Cell Pyroptosis Was Detected by Flow Cytometry Analysis

Cells from each group were collected and counted, washed and resuspended in PBS containing 1 mL of 0.5% BSA, added to the FAM‐FLICA caspase‐1 probe (5 μL, 1:60) and incubated at 37°C in the dark for 60 min. Sample cells were washed with cellular wash buffer and incubated with 500 µl PI staining working solution in the dark for 15 min. Stained HK‐2 cells were analysed using a FACSCalibur flow cytometer (BD Biosciences, San Jose, CA, USA) and Cell‐Quest software.

### 2.12. Statistical Analysis

Graphs were generated using GraphPad Prism 9.0. Statistical analyses were performed with SPSS 21.0 (SPSS, Inc., USA), and data are presented as mean ± standard error of the mean (SEM). Sample sizes were as follows: 15 rats per group for biochemical analyses and western blot; three independent replicates for in vitro experiments. Normality was verified by Shapiro‒Wilk tests (all *p*  > 0.05), with Q–Q plots and histograms confirming normal distribution. The Student’s *t* test was used for two‐group comparisons; one‐way ANOVA followed by Bonferroni’s post hoc test for multiple‐group comparisons (e.g., renal damage scores); Kaplan–Meier survival analysis with log‐rank test for 7‐day rat survival. *p*  < 0.05 was considered statistically significant’.

## 3. Results

### 3.1. Allicin Improved HK‐2 Cell Viability, Kidney Function and Survival in CLP‐Induced Rats

HK‐2 cells and rats were induced with LPS and CLP for 24 h, followed by treatment with allicin and IGF‐1. The objective was to study the effects of allicin on HK‐2 cell activity, kidney function and survival rate in LPS‐induced HK‐2 cells and CLP‐induced rats. Our data showed that LPS stimulation significantly decreased HK‐2 cell viability, as measured by a CCK‐8 assay (Figure 1A). In contrast, allicin significantly increased HK‐2 cell viability induced by LPS (Figure 1A). Similarly, the 7‐day survival rate in the CLP‐induced group was significantly lower (*p* < 0.01; Figure 1B), and serum Scr and BUN levels were markedly elevated in the CLP‐induced group (Figure 1B–D). In addition, allicin improved the 7‐day survival rate (*p* < 0.05; Figure 1B) and decreased the serum levels of Scr and BUN in CLP‐induced rats (Figure 1B–D). Interestingly, our results also revealed that IGF‐1 (a PI3K/AKT agonist) reversed the protective effects of allicin (Figure 1A–D were *p*  < 0.001, *p*  < 0.05, *p*  < 0.001 and *p*  < 0.001 compared with those in the CLP + allicin group). These data indicate that the above effects of allicin may occur through the regulation of the PI3K/AKT pathway, but the specific mechanism needs to be further investigated.

Figure 1Allicin improved HK‐2 cell viability and kidney function and survival in septic rats via the PI3K/AKT pathway both in vitro and in vivo. (A) HK‐2 cell viability was determined by a CCK‐8 assay. (B) The 7‐day survival rate of the rats. (C, D) The serum levels of Scr and BUN were measured by ELISA. Lipopolysaccharide (LPS) (10 µg/mL, 24 h), allicin (A) (50 mg/L in vitro and 30 mg/kg in vivo, 24 h) and IGF‐1 (I) (100 ng/mL in vitro and 5 μg/kg in vivo, 24 h). Control (Con) group; lipopolysaccharide (LPS) group; LPS + allicin (LPS + A) group; LPS + allicin + IGF‐1 (LPS + A + I) group; caecal ligation and puncture (CLP) group; CLP + allicin (CLP + A) group; CLP + allicin + IGF‐1 (CLP + A + I) group. Data are presented as mean ± SEM and were independently analysed in triplicate.  ^∗^ *p* < 0.05,  ^∗∗^ *p* < 0.01 and  ^∗∗∗^ *p* < 0.001.

**Figure:**
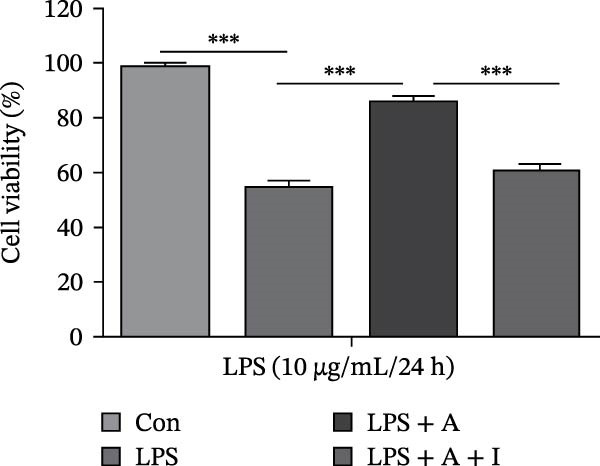
(A)

**Figure:**
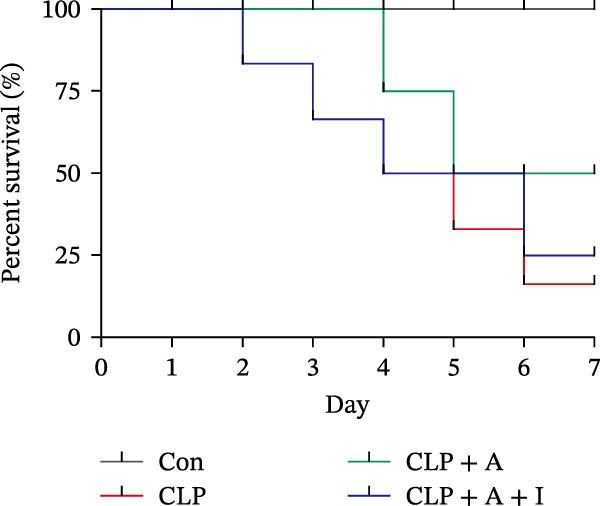
(B)

**Figure:**
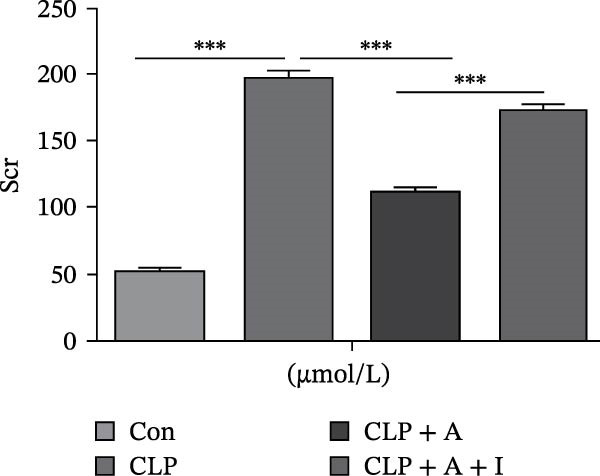
(C)

**Figure:**
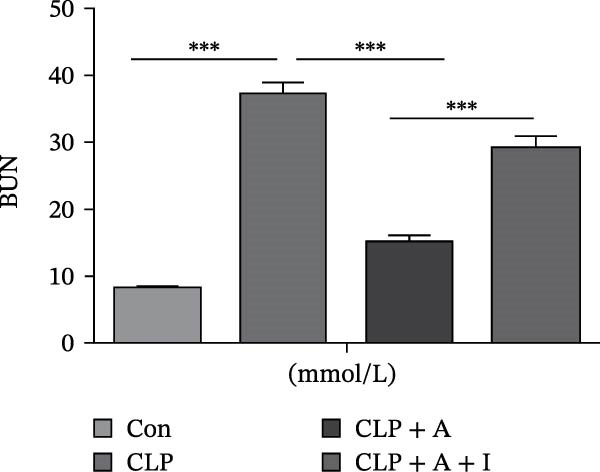
(D)

### 3.2. Allicin Significantly Reduced the Levels of Pyroptosis‐Related Proteins via the PI3K/AKT Pathway In Vivo

To further investigate the effect of allicin on the levels of pyroptosis‐related proteins in CLP‐induced rats, western blotting was performed (Figure 2A,B). Our data show that CLP activated the PI3K/AKT pathway and increased protein expression levels of p‐PI3K, p‐AKT, p‐NF‐κB (p65) and the pyroptosis‐related molecules NLRP3, caspase‐1, and GSDMD‐N in the kidneys of rats in the CLP group (Figure 2A,B). Compared with the CLP group, allicin markedly decreased the protein expression levels of p‐PI3K, p‐AKT, p‐NF‐κB (p65) and the pyroptosis‐related molecules NLRP3, caspase‐1 and GSDMD‐N in the kidneys of rats in the CLP + allicin group (Figure 2A,B). In addition, we also found that the negative regulatory effect of allicin on the above proteins was reversed by IGF‐1 (a PI3K/AKT agonist) in the CLP + Allicin + IGF‐1 group (Figure 2A,B). Overall, these results suggest that allicin may downregulate the expression of pyroptosis‐related proteins by inhibiting the PI3K/AKT pathway, and IGF‐1 may reverse these protective effects by activating the PI3K/AKT pathway.

Figure 2Allicin decreases pyroptosis‐related protein levels through the PI3K/AKT pathway in the kidney tissue of CLP‐induced mice. (A, B) Protein expression was detected by western blotting. Allicin (30 mg/kg, 24 h) and IGF‐1 (5 μg/kg, 24 h). Allicin (A) (30 mg/kg, 24 h) and IGF‐1 (I) (5 μg/kg, 24 h). Control (Con) group; caecal ligation and puncture (CLP) group; CLP + allicin (CLP + A) group; CLP + allicin + IGF‐1 (CLP + A + I) group. The data are presented as the mean ± SEM. The data were independently analysed in triplicate.  ^∗^ *p* < 0.05,  ^∗∗^ *p* < 0.01 and  ^∗∗∗^ *p* < 0.001.

**Figure:**
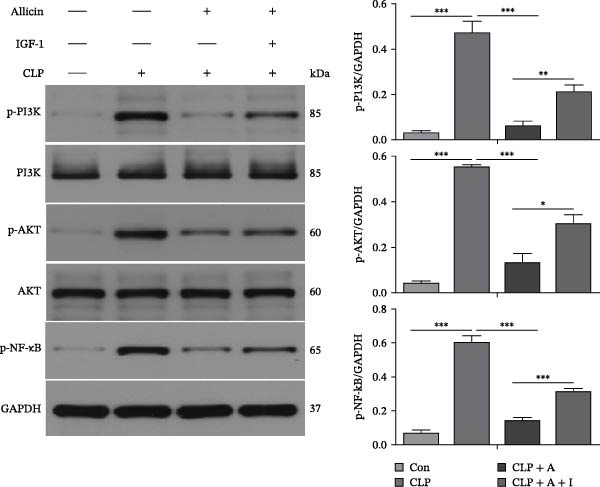
(A)

**Figure:**
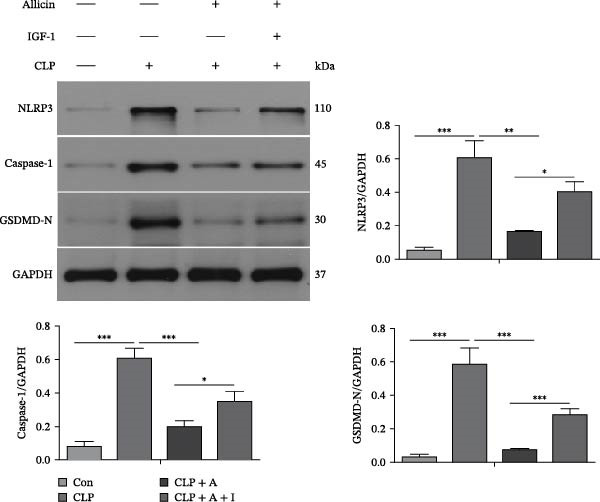
(B)

### 3.3. Allicin Ameliorates Kidney Injury by Negatively Regulating the PI3K/AKT Pathway in CLP‐Induced Rats

**Figure 3:**
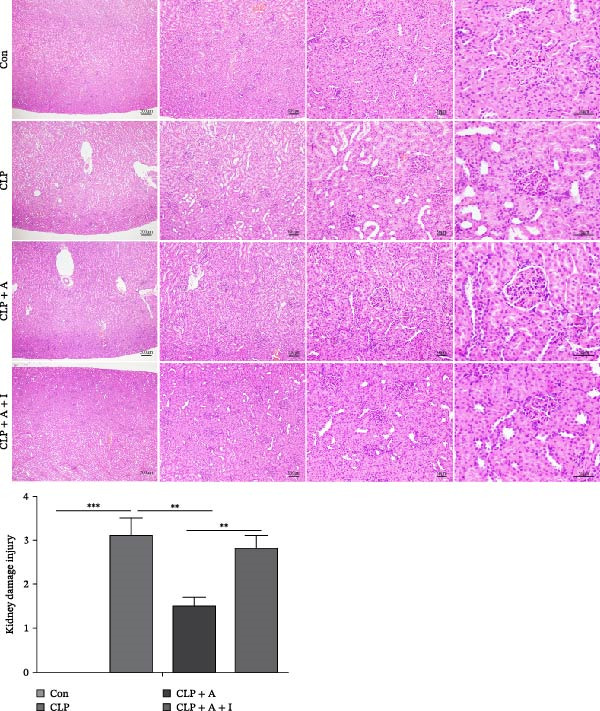
Allicin ameliorated kidney injury via the PI3K/AKT pathway in CLP‐induced rats. Kidney tissue was analysed by HE staining (40×, 100×, 200× and 400×). Allicin (A) (30 mg/kg, 24 h), IGF‐1 (I) (5 μg/kg, 24 h). Control (Con) group; caecal ligation and puncture (CLP) group; CLP + allicin (CLP + A) group; CLP + allicin + IGF‐1 (CLP + A + I) group. The data are presented as the mean ± SEM. The data were independently analysed in triplicate.  ^∗^ *p* < 0.05,  ^∗∗^ *p* < 0.01 and  ^∗∗∗^ *p* < 0.001.

In this section, the effects of allicin on S‐AKI and whether allicin acts through the PI3K/AKT pathway were explored. Kidney histopathological damage was assessed by HE staining. CLP caused significant tissue oedema, tubular dilatation and vacuolar degeneration of tubular epithelial cells in the CLP group (*p* < 0.001 compared with those in the control group). Moreover, administration of allicin significantly improved histopathological damage in the CLP + allicin group (*p* < 0.01 compared with the CLP group). Conversely, compared with allicin alone, IGF‐1 attenuated the protective effect of allicin against CLP‐induced kidney injury (*p* < 0.01 compared to CLP + allicin group). Thus, we hypothesised that allicin exerts the above effects via the PI3K/AKT pathway (Figure 3).

### 3.4. Allicin Inhibits Kidney Tissue Pyroptosis Through the PI3K/AKT Pathway in CLP‐Induced Rats

To further investigate the effect of allicin on CLP‐induced renal tissue pyroptosis, ELISA and TUNEL/caspase‐1 immunofluorescence double staining were performed in rats. CLP significantly increased the levels of IL‐1β and IL‐18 in the CLP group, as measured by ELISA (Figure 4A). In contrast, treatment with allicin significantly reduced the levels of IL‐1β and IL‐18 in the CLP + allicin group (Figure 4A). In addition, numerous pyroptotic cells (green and red double staining) were observed in the kidney tissue of the CLP group (Figure 4B). Moreover, allicin significantly improved CLP‐induced pyroptosis in the kidney tissue of the CLP + allicin group (Figure 4B). IGF‐1 intervention was used to investigate whether allicin plays a regulatory role through the PI3K/AKT pathway. Interestingly, administration of IGF‐1 (a PI3K/AKT agonist) antagonised the protective effect of allicin on CLP‐induced renal tissue pyroptosis and the negative regulation of inflammatory factors (IL‐1β and IL‐18). The above data suggest that allicin inhibits CLP‐induced kidney pyroptosis through the PI3K/AKT pathway.

Figure 4Allicin inhibited CLP‐induced renal pyroptosis through the PI3K/AKT pathway. (A) Inflammatory factors (IL‐1β and IL‐18) were measured by ELISA. (B) Pyroptosis in kidney tissue was assessed by TUNEL (green) and caspase‐1 (red) immunofluorescence double staining (400×). Double‐stained cells (%) = number of double‐stained positive cells/(TUNEL‐positive cells + caspase‐1‐positive cells‐double‐stained positive cells) × 100%. Allicin (A) (30 mg/kg, 24 h) and IGF‐1 (I) (5 μg/kg, 24 h). Control (Con) group; caecal ligation and puncture (CLP) group; CLP + allicin (CLP + A) group; CLP + allicin + IGF‐1 (CLP + A + I) group. The data are presented as the mean ± SEM. The data were independently analysed in triplicate.  ^∗^ *p*  < 0.05,  ^∗∗^ *p*  < 0.01 and  ^∗∗∗^ *p*  < 0.001.

**Figure:**
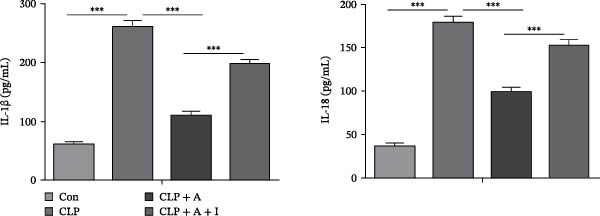
(A)

**Figure:**
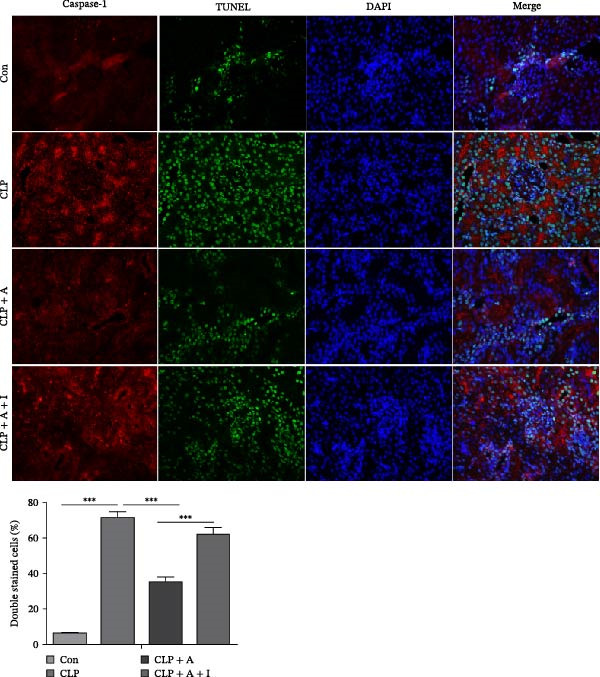
(B)

### 3.5. Allicin Significantly Decreased the Expression Levels of Pyroptosis‐Related Proteins in LPS‐Induced HK‐2 Cells

To explore the effects of allicin on the levels of pyroptosis‐related proteins in LPS‐induced HK‐2 cells, western blotting was performed in vitro (Figure 5). As with the in vivo studies, western blotting revealed that LPS significantly increased the protein levels of p‐PI3K, p‐AKT, p‐NF‐κB (p65) and the pyroptosis‐related molecules NLRP3, caspase‐1 and GSDMD‐N in HK‐2 cells from the LPS group (Figure 5A,B). Furthermore, allicin markedly reversed the LPS‐induced upregulation of p‐PI3K, p‐AKT, p‐NF‐κB (p65) and the pyroptosis‐related molecules NLRP3, caspase‐1, and GSDMD‐N in the LPS + allicin group (Figure 5A,B). Conversely, our data revealed that the negative regulatory effect of allicin on the above proteins could be antagonised by IGF‐1 (a PI3K/AKT agonist) in the LPS + allicin + IGF‐1 group (Figure 5A,B). Overall, these data suggest that allicin may downregulate pyroptosis‐related proteins by inhibiting the PI3K/AKT pathway and that IGF‐1 may reverse the above protective effects of allicin through PI3K/AKT activation.

Figure 5Allicin decreased the levels of pyroptosis‐related proteins in LPS‐treated HK‐2 cells. (A, B) Protein expression was detected by western blotting. Lipopolysaccharide (LPS) (10 µg/mL, 24 h), allicin (A) (50 mg/L, 24 h) and IGF‐1 (I) (100 ng/mL, 24 h). Control (Con) group; lipopolysaccharide (LPS) group; LPS + allicin (LPS + A) group; LPS + allicin + IGF‐1 (LPS + A + I) group. The data are presented as the mean ± SEM. The data were independently analysed in triplicate.  ^∗^ *p* < 0.05,  ^∗∗^ *p* < 0.01 and  ^∗∗∗^ *p* < 0.001.

**Figure:**
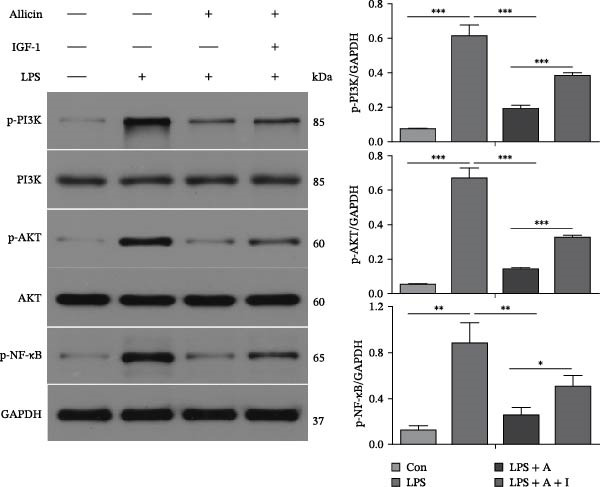
(A)

**Figure:**
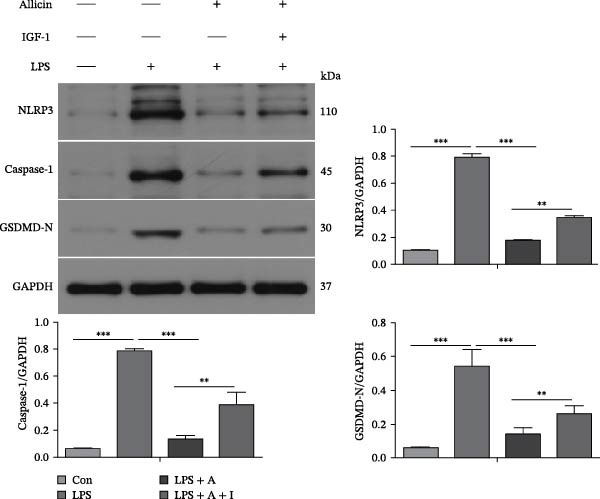
(B)

### 3.6. Allicin Inhibited Pyroptosis in LPS‐Induced HK‐2 Cells via the PI3K/AKT Pathway

We performed ELISA, PI staining and flow cytometry analysis on HK‐2 cells. We aimed to investigate the effect of allicin on LPS‐induced pyroptosis in HK‐2 cells in vitro. ELISA data revealed that LPS stimulation significantly increased the levels of IL‐1β and IL‐18 and the LDH release rate in LPS‐induced HK‐2 cells (Figure 6A). In contrast, allicin clearly reduced the levels of IL‐1β and IL‐18 and the LDH release rate in LPS‐induced HK‐2 cells (Figure 6A). Similarly, numerous pyroptotic HK‐2 cells were observed in the LPS‐induced HK‐2 cells (Figure 6B,C). Moreover, allicin significantly improved LPS‐induced pyroptosis in HK‐2 cells in the LPS + allicin group (Figure 6B,C). IGF‐1 intervention was used to investigate whether allicin plays a regulatory role through the PI3K/AKT pathway. As with the in vivo studies, the administration of IGF‐1 (a PI3K/AKT agonist) antagonised the above regulatory effects of allicin (Figure 6A–C). In summary, these data suggest that allicin inhibits pyroptosis via the PI3K/AKT pathway in LPS‐induced HK‐2 cells.

Figure 6Allicin inhibited pyroptosis in LPS‐induced HK‐2 cells via the PI3K/AKT pathway. (A) Inflammatory factor (IL‐1β and IL‐18) levels and the LDH release rate were detected by ELISA. (B, C) Pyroptosis of HK‐2 cells was measured by PI staining and flow cytometry analysis. PI‐positive cells (red fluorescent cells) and DAPI‐positive cells (blue fluorescent cells). Pyroptosis rate (%) = the number of PI‐positive cells/the number of DAPI‐positive cells × 100%. Lipopolysaccharide (LPS) (10 µg/mL, 24 h), allicin (A) (50 mg/L, 24 h) and IGF‐1 (I) (100 ng/mL, 24 h). Control (Con) group; lipopolysaccharide (LPS) group; LPS + allicin (LPS + A) group; LPS + allicin + IGF‐1 (LPS + A + I) group. The data are presented as the mean ± SEM. The data were independently analysed in triplicate.  ^∗^ *p* < 0.05, 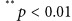 and  ^∗∗∗^ *p* < 0.001.

**Figure:**
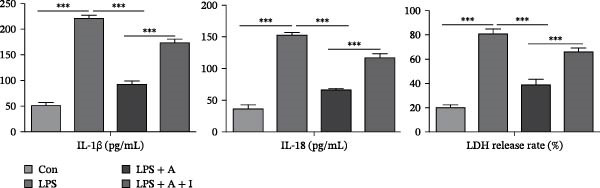
(A)

**Figure:**
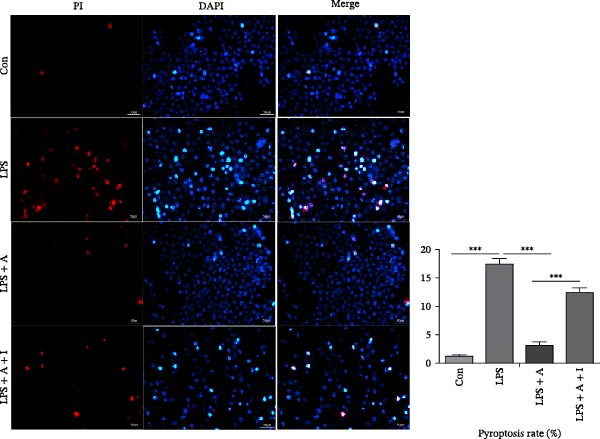
(B)

**Figure:**
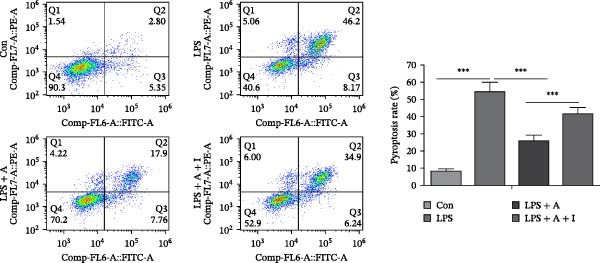
(C)

## 4. Discussion

In this study, we demonstrate that allicin alleviates S‐AKI by inhibiting pyroptosis via negative regulation of the PI3K/AKT pathway. Our findings provide new insights into the protective mechanisms of allicin, which has been previously studied for its anti‐inflammatory and organ‐protective effects in sepsis [13, 14], and further expand its therapeutic targets from oxidative stress/apoptosis to pyroptosis.

Notably, prior research has shown that allicin protects against renal ischaemia‒reperfusion injury by reducing oxidative stress and apoptosis [28, 29], while our data identify pyroptosis as a novel target of allicin in S‐AKI: in LPS‐stimulated HK‐2 cells, allicin significantly reduced NLRP3/caspase‐1/GSDMD‐N expression (Figure 5B), which mirrors the report by Sun et al. [26] that allicin inhibits NLRP3 in LPS‐induced cardiomyocyte injury. This consistency suggests that NLRP3 may be a conserved target of allicin in inflammatory organ damage, but our study extends this finding to S‐AKI and links it to the PI3K/AKT pathway. In a mechanistic parallel, Qian et al. [23] reported that allicin suppresses the PI3K/AKT/NF‐κB pathway to alleviate osteoarthritis, and our results confirm this regulatory axis in S‐AKI—specifically, we found that IGF‐1 (a PI3K/AKT agonist) reversed allicin’s inhibitory effects on p‐PI3K/p‐AKT and pyroptosis (Figures 2A, 4B, 5A), directly validating that PI3K/AKT is a key mediator of allicin’s anti‐pyroptotic role.

Our results also complement and extend emerging evidence on pyroptosis in S‐AKI. Ye et al. [30] reported that caspase‐11‐mediated non‐classical pyroptosis contributes to tubular injury, while we focused on the classical NLRP3‐caspase‐1‐GSDMD pathway and showed that allicin reduces IL‐1β/IL‐18 release (Figures 4A, 6A) by blocking this pathway—this fills the gap in understanding how allicin regulates classical pyroptosis in S‐AKI. Additionally, Sun et al. [31] found that GSDMD‐N‐driven inflammation exacerbates S‐AKI, and our data further show that allicin downregulates GSDMD‐N via PI3K/AKT inhibition (Figures 2B, 5B), providing a clear mechanistic link between allicin’s pathway regulation and pyroptosis execution.

This study focuses on the classical NLRP3‐caspase‐1‐GSDMD pyroptosis pathway and does not involve non‐classical pathways or other effector molecules. Given that caspase‐11 mediates renal tubular pyroptosis in sepsis [30], future studies could use western blotting and immunofluorescence co‐localisation to detect the effects of allicin on the expression and cleavage of caspase‐11 and the activation of downstream GSDMD. For GSDME [32], the level of GSDME‐N in renal tissues/cells could be measured, and IGF‐1 could be used to verify whether the PI3K/AKT pathway is involved. Additionally, PI3Kγ inhibitors [22] and overexpression plasmids could be employed to identify the specific PI3K isoform targeted by allicin.

## 5. Conclusions

In summary, our in vivo and in vitro results revealed that allicin alleviated CLP‐ or LPS‐induced pyroptosis, improved kidney function, and increased the 7‐day survival rate and HK‐2 cell activity. These effects were accompanied by downregulation of p‐PI3K and p‐AKT in kidney tissue and HK‐2 cells. The protective effect of allicin on S‐AKI was blocked by IGF‐1, indicating that allicin may attenuate S‐AKI by inhibiting inflammation and pyroptosis via the PI3K/AKT pathway. These results contribute to the development of new therapeutic strategies for S‐AKI. However, it is critical to emphasise that these findings are derived from preclinical models, and clinical translation requires validation through human trials, including dose optimisation, pharmacokinetic evaluations, and long‐term safety assessments. Additionally, mechanistic confirmation in human sepsis‐AKI cohorts is essential to validate the PI3K/AKT pathway as a therapeutic target. Although further studies are needed to fully clarify the specific mechanisms involved, translational research remains a prerequisite before the clinical application of allicin can be considered.

## Author Contributions

Yanlei Zheng performed the experiments, analysed the data, prepared the figures and wrote the manuscript. Shi Li and Li Zhu performed the experiments and interpreted the data. Li Zhang provided laboratory space and funding, designed the study and analysed the data.

## Funding

This work was financially supported by the Research Projects of the Biomedical Center of Hubei Cancer Hospital (Grant 2022SWZX26).

## Disclosure

All authors have read and approved the final manuscript.

## Ethics Statement

The animal experiments involved in this study were approved by the Ethics Committee of the Huazhong University of Science and Technology (Approval Number HUST‐2023‐ICUC‐406). All experiments were performed in accordance with the relevant guidelines and regulations.

## Conflicts of Interest

The authors declare no conflicts of interest.

## Data Availability

The datasets generated during the current study are available from the corresponding author upon reasonable request.
